# Hepatoprotective effect of licorice, the root of *Glycyrrhiza uralensis* Fischer, in alcohol-induced fatty liver disease

**DOI:** 10.1186/s12906-016-0997-0

**Published:** 2016-01-22

**Authors:** Jae-Chul Jung, Yun-Hee Lee, Sou Hyun Kim, Keuk-Jun Kim, Kyung-Mi Kim, Seikwan Oh, Young-Suk Jung

**Affiliations:** 1 Life Science Research Institute, Novarex Co., Ltd, Ochang, Cheongwon, Chungbuk 363-885 Republic of Korea; 2 College of Pharmacy, Yonsei University, Incheon, 406-840 Republic of Korea; 3 College of Pharmacy, Pusan National University, Busan, 609-735 Republic of Korea; 4 Department of Biomedical Laboratory Science, Daekyeung College, Gyeongsan, 712-719 Republic of Korea; 5 Department of Molecular Medicine and Tissue Injury Defense Research Center, School of Medicine, Ewha Woman’s University, Seoul, 158-710 Republic of Korea

**Keywords:** Licorice, Alcohol-induced liver injury, Glutathione, TNF-α

## Abstract

**Background:**

Our previous study suggested that licorice has anti-inflammatory activity in lipopolysaccharide-stimulated microglial cells and anti-oxidative activity in *tert*-butyl hydroperoxide–induced oxidative liver damage. In this study, we evaluated the effect of licorice on chronic alcohol-induced fatty liver injury mediated by inflammation and oxidative stress.

**Methods:**

Raw licorice was extracted, and quantitative and qualitative analysis of its components was performed by using LC–MS/MS. Mice were fed a liquid alcohol diet with or without licorice for 4 weeks.

**Results:**

We have standardized 70 % fermented ethanol extracted licorice and confirmed by LC-MS/MS as glycyrrhizic acid (GA), 15.77 ± 0.34 μg/mg; liquiritin (LQ), 14.55 ± 0.42 μg/mg; and liquiritigenin (LG), 1.34 ± 0.02 μg/mg, respectively. Alcohol consumption increased serum alanine aminotransferase and aspartate aminotransferase activities and the levels of triglycerides and tumor necrosis factor (TNF)-α. Lipid accumulation in the liver was also markedly induced, whereas the glutathione level was reduced. All these alcohol-induced changes were effectively inhibited by licorice treatment. In particular, the hepatic glutathione level was restored and alcohol-induced TNF-α production was significantly inhibited by licorice.

**Conclusion:**

Taken together, our data suggests that protective effect of licorice against alcohol-induced liver injury may be attributed to its anti-inflammatory activity and enhancement of antioxidant defense.

**Electronic supplementary material:**

The online version of this article (doi:10.1186/s12906-016-0997-0) contains supplementary material, which is available to authorized users.

## Background

**Fig. 1:**
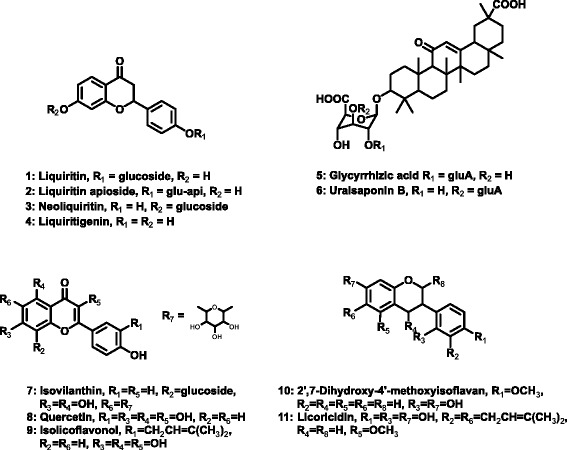
Structures of Liquiritins (1 ~ 4), Glycyrrhizic acids (5 ~ 6), and Flavones (7 ~ 11) in licorice

Licorice is the root of *Glycyrriza uralensis* Fischer, *Glycyrrhiza glabra* Linné or *Glycyrrhiza inflata* Batalin (Fabaceae), which has been used as traditional medicine since ancient times. In particular, licorice was used as a medical raw material for multiple purposes such as antidote, antitussive expectorant, relaxant, to relieve pain that occurs because of a sudden nervous breakdown of muscle or tissue, to reduce weight gain, to increase white blood cell count, and also because of its diuretic and anti-inflammatory effects [1]. Although licorice has been used in both Eastern and Western medicine to treat a wide variety of diseases from common cold to liver disease, more scientific evidence is needed to prove its potential preventive and therapeutic benefits. The biologically active components of licorice are liquiritins (LQ), liquiritigenin (LG), glycyrrhizic acids (GAs), and flavones (Fig. 1). Various biological effects of these compounds and pharmacokinetics of glycyrrhizic acid have been reported [2–4]. In addition, studies have been performed to analyze and characterize primary and secondary metabolites of licorice [5, 6].

Accumulating lines of evidence show that licorice has anti-inflammatory, anticancer, antioxidant, and anti-microbial effects [1, 4, 7–9]. In particular, recent studies on hepatoprotective effects of licorice suggest that it can reduce liver injury by enhancing antioxidant and anti-inflammatory capacity [7, 10]. Administration of licorice extract prevented CCl_4_-induced hepatotoxicity by increasing antioxidant enzyme activity and decreasing TNF-α production [11]. Jung et al. [12] investigated the hepatoprotective effects of 18β-glycyrrhetinic acid, one of the active compounds in licorice, in a CCl_4_-induced liver injury model. Treatment with 18β-glycyrrhetinic acid inhibited the increase in serum alanine aminotransferase (ALT) and aspartate aminotransferase (AST) activities and hepatic lipid peroxidation in a dose-dependent manner. In addition, 18β-glycyrrhetinic acid significantly protected against glutathione (GSH) depletion. Although these studies show a promising effect of licorice in preventing liver injury, their limitation was that the chemically induced acute hepatotoxicity model used was not very relevant to clinical situations.

Alcohol abuse causes a range of acute and chronic health problems worldwide, which lead to morbidity and mortality. Depending on overall alcohol consumption and drinking patterns, chronic exposure to alcohol is harmful to the central nervous system and many organs, including the liver. Among alcohol-induced liver diseases, fatty liver is the most common histopathologic condition in drinkers. Although alcohol-induced fatty liver is widely considered to be benign and to have a very low risk of progression, clinical studies have provided evidence that it is an important pathogenic factor in the development of liver disease [13–15]. Specifically, the authors suggested that both oxidative stress and inflammation as second hits are critical factors in the pathological progression from simple fat accumulation to liver disease. Recently, we reported that licorice extract had an anti-inflammatory effect in lipopolysaccharide-stimulated microglial cells and acted as an antioxidant in a *tert*-butyl hydroperoxide–induced oxidative liver injury model [16]. Therefore, it was of interest to examine the effects of licorice on chronic alcohol-induced fatty liver, which is more relevant to clinical situations. In this study, we examined the preventive effect of licorice in alcoholic fatty liver by administering its extract to mice exposed to alcohol for 4 weeks.

## Methods

### Extraction

**Fig. 2:**
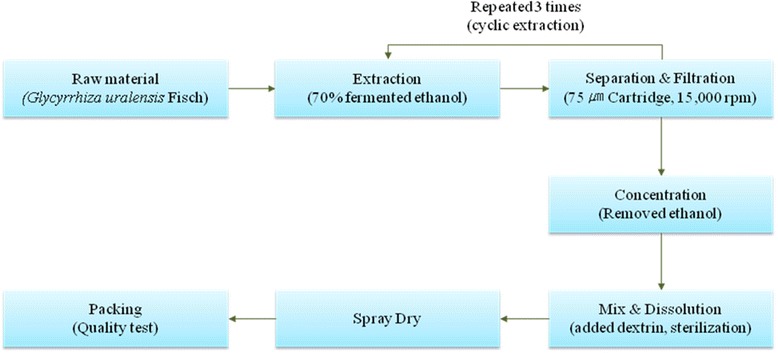
Manufacture process for production of licorice extracted powder

*Glycyrrhiza uralensis* Fisher (Fabaceae) was cultivated in Jecheon, Chungbuk Province, Korea. The raw material has been provided by the Korea Licorice Farming Association in 2013 and its extraction was produced by Tecos Co., Ltd (Chuncheon, Korea). Prof. Min Hye Yang of the Pusan National University identified plant material and a voucher specimen (PNU-0020) has been deposited in the Medicinal Herb Garden, Pusan National University (Busan, Korea). The analysis of biological component and microbiological test were confirmed by Novarex Co., Ltd (Ochang, Korea). All other chemicals were purchased from Sigma Chemical Co. (St. Louis, MO, USA) and Wako Pure Chemical Industries (Osaka, Japan). The raw material of licorice (the root of *Glycyrrhiza uralensis* Fisher, 400 kg) was extracted for 3 h using a reflux circulation of 70 % aqueous ethanol (2800 L). The extracts was cooled at 30 ~ 35 °C and filtered using 75 μm cartridge and then the residue of raw materials was removed through subject of a centrifuge. The residue was concentrated *in vacuo* under reduced pressure (10 atm, 55 ~ 58 °C) to reach 10 ~ 20 brix materials (52 ~ 64 kg). The residue was blended with dextrin and sterilized at 95 °C for 30 min and then it was spray-dried (liquid temperature: 75 ~ 80 °C, the blowing temperature of 180 °C, atomizer 18,000 rpm) to provide a licorice extract powder (90 kg, 11.3 %). To establish bulk scale production of licorice extracts, we confirmed manufacturing process based on experimental pilot condition using Jecheon domestic licorice in Korea (Fig. 2).

### Analysis of licorice extract

**Fig. 3:**
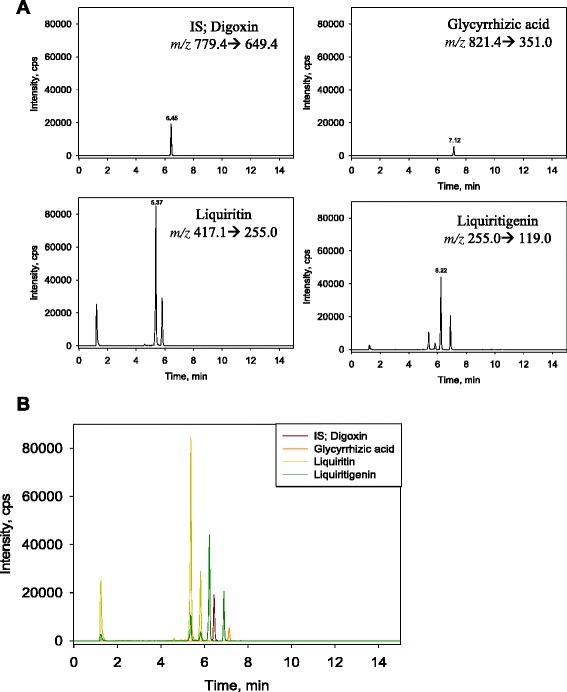
LC-MS/MS spectrum of standard (**a**) and licorice extract (**b**)

To confirm two index components such as triterpenoid saponin series GA and flavonoids LQ, we performed quantitative and qualitative analysis through HPLC and HPLC-MS/MS based on United States Pharmacopoeia and Korean Pharmacopeia as standard analytical methods (Fig. 3).

### Analytical condition of LC-MS/MS

We used digoxin as internal standard in order to quantitative analysis of major components GA, LQ, and LG of licorice extract. In addition, we performed material separation for each component of the material using LUNA C_18_ column (2.0 × 150 mm, 5 μm). Solvent A was water with 1.0 % acetic acid and solvent B was acetonitrile with 1.0 % acetic acid. The gradients of solvents were as followings: 0 min, 10 % B; 1 min, 10 % B; 6.5 min, 90 % B; 8 min, 90 % B; 8.5 min, 10 % B; 15 min, 10 % B. Samples were dissolved in 50 % acetonitrile and the injection volume of each sample was 5 μl. Detailed condition for LC-MS/MS analysis is in the Table 1.

**Table 1:** Condition for LC-MS/MS analysis of Licorice extracts

A.	
HPLC Condition	
Column	Luna C_18_ RP column (2.0 × 150 mm, 5 μm)
Flow rate	0.3 mL/min
Injection volume	5 μL
Column temperature	40 °C
Autosampler temperature	4 °C
B.	
Mass condition	
Ion source	Turbo spray (Negative)
Curtain Gas	30 psi
Collision Gas	N_2_ (Medium)
Ionspray Voltage	- 4.0 kV
Source temperature	400 °C
Gas 1	40 psi
Gas 2	50 psi

### Animals and treatments

Male C57BL/6 mice were purchased from Orient Bio (Sungnam, Korea). The use of animals was in compliance with the guidelines established and approved by the Institutional Animal Care and Use Committee in Pusan National University (PNU-2014-0568). Animals were acclimated to temperature (22 ± 2 °C) and humidity (55 ± 5 %) controlled rooms with a 12-h light/dark cycle for 1 week prior to use. The diets were purchased from Dyets Inc. (Bethlehem, PA, USA). Mice were fed a Lieber–DeCarli liquid diet with or without ethanol for 4 week. For the control liquid diet, 35 % of energy was derived from fat, 18 % from protein, and 47 % from carbohydrates; the liquid ethanol diet contained 35 % of energy from fat, 18 % from protein, 11 % from carbohydrates, and 36 % from ethanol.

### Serum biochemistry and histopathologic evaluation

Serum levels of alanine aminotransferase (ALT), aspartate aminotransferase (AST), total serum triglyceride (TG) were measured using Automated Chemistry Analyzer (Prestige 24I; Tokyo Boeki Medical System, Tokyo, Japan). For histopathologic evaluation, a cross section of the left lateral lobe of the liver was sliced at 10 μm, immersed in propylene glycol for 5 min, and stained with Oil red O for 7 min. After rinsing with 85 % propylene glycol and distilled water, the sections were counterstained with hematoxylin for 2 min before microscopic examination.

### Measurement of serum TNF-α

The levels of serum TNF-α were determined by enzyme-linked immunosorbent assay using a commercially available kit (R&D Systems, Minneapolis, MN) according to the manufacturer’s instruction.

### Determination of hepatic triglyceride contents

Total lipids of the liver were extracted from homogenate prepared from 100 mg of mouse liver using the mixture of chloroform/methanol (2:1, v/v). Triglycerides in total lipid were determined enzymatically using a commercially available enzymatic kit (Sigma Chemical Co.) according to the manufacturer’s protocol.

### Measurement of hepatic glutathione (GSH)

Liver was homogenized in a four-fold volume of ice-cold 1 M perchloric acid. After the denatured protein was removed by centrifugation at 10,000 g for 10 min, the supernatant was assayed for the total GSH concentration using a HPLC separation/fluorometric detection method [17].

### Real time RT-PCR

Total RNA was purified from liver tissue using the RNeasy kit (Qiagen, Valencia, CA, USA). cDNA synthesis was accomplished with iScript™ cDNA Synthesis system (Bio-Rad, Hercules, CA, USA). Real time RT-PCR was performed using Thunderbird SYBR qPCR mix (Toyobo Co., Ltd., Osaka, Japan) according to the manufacturer’s protocol. Relative values of gene expression were normalized to 18S ribosomal RNA. Primer sequences and full name of the genes are provided in Additional file 1: Table S1.

### Statistical analysis

All results expressed as mean ± s.d. were analyzed by one-way analysis of variance (ANOVA) followed by Newman-Keuls multiple range test (parametric). The acceptable level of significance was established at *P* < 0.05.

## Results

We analyzed licorice extract composition based on testing condition using a Shiseido HPLC system coupled to an AB Sciex electrospray-ionization (ESI) mass-spectrometer (Table 2). For quantitative LC-MS/MS analysis of each component, we established optimal conditions for the precursor ion and product ion by adjusting collision energy, cone voltage, and cone temperature of the ion source (Table 1). The instrument was operated in the multiple-reaction-monitoring (MRM) mode.

**Table 2:** Linearities, regression equation, correlation coefficients, limit of detection (LOD), and limit of quantitation (LOQ) for glycryrrhizic acid (GA), liquiritin (LQ) and liquiritigenin (LG)

Compounds	Linear range (ng/ml)	Regression equation^a^	Correlation coefficient	LOD^b^ (ng/ml)	LOQ^c^ (ng/ml)
Glycyrrhizic acid (GA)	12.5–5000	Y = 0.0004x – 0.0055	0.9999	4.62	13.99
Liquiritin (LQ)	12.5–1000	Y = 0.0059x + 0.0451	0.9997	1.27	3.86
Liquiritigenin (LG)	12.5–500	Y = 0.0289x + 0.1574	0.9998	0.54	1.64

^a^y: Analyte area / IS area; x: concentration (ng/mL) of compounds

^b^LOD = 3.3 x δ /S (δ; standard deviation, S; slope of the calibration curve)

^c^LOQ = 10 x δ /S (δ; standard deviation, S; slope of the calibration curve)

We prepared the calibration curves for GA, LQ, and LG according to the concentration-dependent ESI method in LC-MS/MS analysis. We found that the correlation coefficient (r^2^) values were 0.9999, 0.9997, and 0.9998, respectively, which showed good linearity of the calibration curves (Table 2). The limit of detection (LOD) was 4.29 ng/mL, 1.27 ng/mL, and 0.54 ng/mL, whereas the limit of quantitation (LOQ) was 13.99 ng/mL, 3.86 ng/mL, and 1.64 ng/mL, respectively.

We performed quantitative analysis of GA, LQ, and LG. The MRM conditions were m/z 821.4 (precursor ion) → 351.0 (product ion) for GA, m/z 417.1 (precursor ion) → 255.0 (product ion) for LQ, and m/z 255.0 (precursor ion) → 119.0 (product ion) for LG. We set up the amount of digoxin as internal standard at m/z 779.4 (precursor ion) → 649.4 (product ion). For quantitative analysis, we used the calibration curves to calculate the ratios of compounds in the analyzed material to respective standards. We diluted licorice extract 1/20 to ensure that the concentrations of its components are within the quantitative ranges of the calibration curves, and then multiplied the obtained concentrations by 20 (the dilution factor). The results of quantitation of the licorice extract components were as follows: GA, 15.77 ± 0.34 μg/mg; LQ, 14.55 ± 0.42 μg/mg; and LG, 1.34 ± 0.02 μg/mg (Table 3).

**Table 3:** Analytical LC-MS/MS data of licorice extracts

Compounds		Peak area	IS area	Analytes/IS ratio	Calculation	μg/mg	Ave.	S.D.
Glycyrrhizic Acid (GA)	1/20 dil.1	23,132	74,555	0.31	789.42	15.79	15.77	0.34
	1/20 dil.2	23,226	73,402	0.32	804.80	16.10		
	1/20 dil.3	23,016	75,956	0.30	771.29	15.43		
Liquiritin (LQ)	1/20 dil.1	316,205	74,555	4.24	711.21	14.22	14.55	0.42
	1/20 dil.2	328,810	73,402	4.48	751.61	15.03		
	1/20 dil.3	326,100	75,956	4.29	720.03	14.40		
Liquiritigenin (LG)	1/20 dil.1	153,592	74,555	2.06	65.84	1.32	1.34	0.02
	1/20 dil.2	154,374	73,402	2.10	67.33	1.35		
	1/20 dil.3	159,391	75,956	2.10	67.16	1.34		

**Fig. 4:**
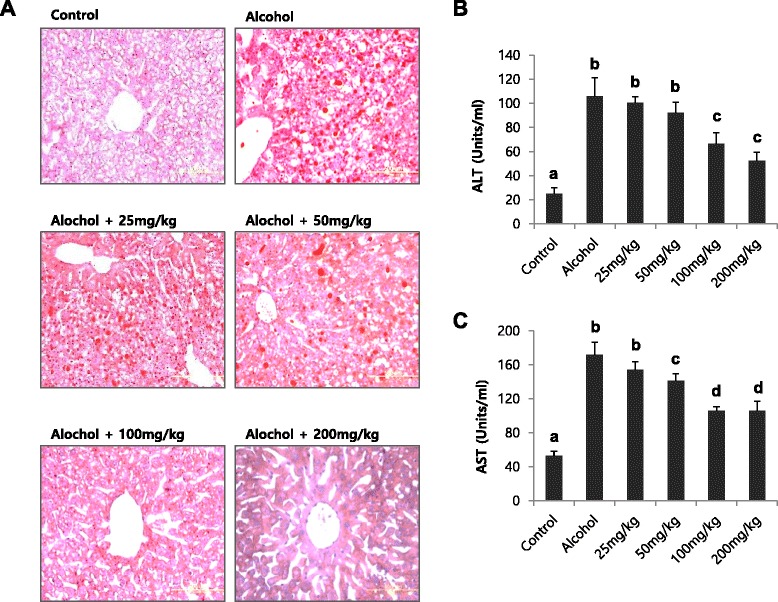
Dose-dependence study of licorice in alcohol-induced fatty liver. Hepatic lipid accumulation (**a**), serum ALT (**b**) and AST (**c**) activities in mice fed different diet with or without licorice. The livers were stained with Oil red O for histopathological examination. Each value represents the mean ± SD for 6 mice. Values with different letters are significantly different from one another (ANOVA followed by Newman-Keuls multiple range test *P* < 0.05)

To test the effect of licorice on alcohol-induced fatty liver, a dose-dependence study was performed in mice fed a standard Lieber–DeCarli liquid diet supplemented with ethanol for 4 weeks (Fig. 4). Different dosage of licorice ranging from 25 mg/kg body weight to 200 mg/kg body weight was orally administered every day from the beginning of the liquid diet. At the end of the treatment period, lipid accumulation in the liver was evaluated by Oil Red-O staining (Fig. 4a). Serum ALT and AST activities were also determined (Fig. 4b and c). The dose of 25 to 50 mg/kg body weight was not effective, but treatment with doses exceeding 100 mg/kg significantly reversed hepatic lipid accumulation and serum ALT, AST activities (Fig. 4).

**Fig. 5:**
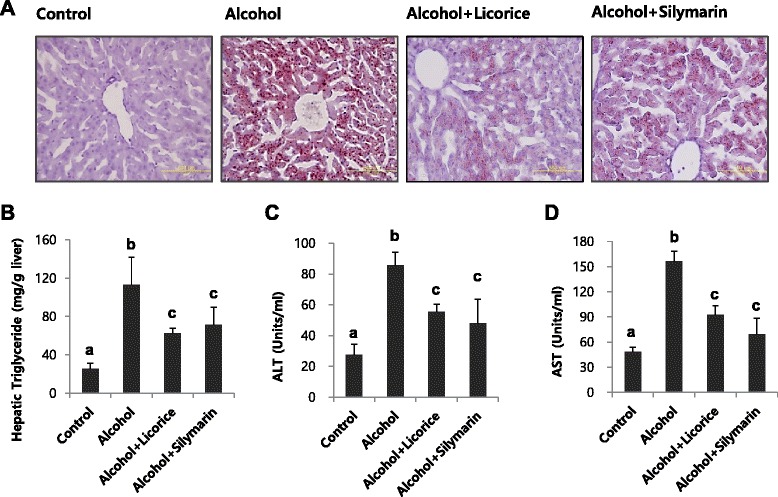
Effect of licorice on hepatic lipid accumulation and serum ALT, AST compared with silymarin as a positive control. Hepatic lipid accumulation (**a**) and triglyceride level (**b**), serum ALT (**c**) and AST (**d**) activities in mice fed different diet with or without licorice. The livers were stained with Oil red O for histopathological examination. Each value represents the mean ± SD for 6 mice. Values with different letters are significantly different from one another (ANOVA followed by Newman-Keuls multiple range test *P* < 0.05)

We compared the effect of licorice (100 mg/kg body weight) on alcoholic fatty liver with that of silymarin (100 mg/kg body weight; Sigma Chemical Co., Cat S0292), which is a well-known compound that alleviates alcohol-induced liver injury. Using staining with Oil Red-O and evaluation of hepatic triglyceride, we found that licorice was a promising candidate to alleviate alcoholic fatty liver in comparison with the inhibitory effect of silymarin on fat accumulation induced by chronic alcohol ingestion (Fig. 5a and b).

**Fig. 6:**
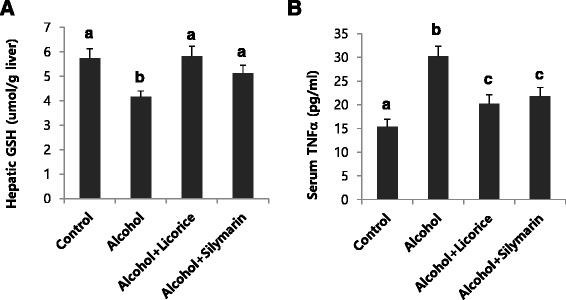
Effect of licorice on the level of hepatic GSH (**a**) and serum TNFα (**b**) in alcohol-induced fatty liver. Each value represents the mean ±SD for 6 mice. Values with different letters are significantly different from one another (ANOVA followed by Newman-Keuls multiple range test *P* < 0.05)

Biochemical analyses of serum ALT, AST activities, and liver triglyceride levels corresponded to the histopathologic findings: licorice administration significantly attenuated the effects of ethanol on triglyceride accumulation in the liver and ALT and AST activities in serum (Fig. 5c and d). The hepatic GSH content in ethanol-treated mice was significantly lower than that in the control mice. Licorice treatment restored GSH to its original level (Fig. 6a). Licorice also significantly decreased the level of serum TNF-α (Fig. 6b).

**Fig. 7:**
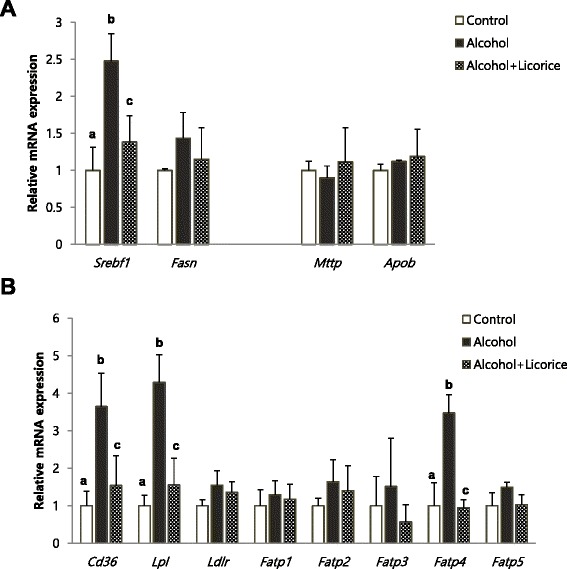
Effect of licorice on the level of hepatic mRNA expression in alcohol-induced fatty liver. mRNA expression of *Srebf1* and *Fasn* for lipogenesis, and *Mttp* and *Apob* for lipid export in the liver (**a**). Expression of mRNA related to lipid uptake in the liver (**b**). Each value represents the mean ± SD for 6 mice. Values with different letters are significantly different from one another (ANOVA followed by Newman-Keuls multiple range test *P* < 0.05)

To find detailed mechanism for the hepatic lipid lowering effect of licorice, we analyzed the hepatic expression levels of the lipogenic genes such as *Srebf1* and *Fasn*, and the genes involved in lipid transportation such as *Mttp, Apob, Cd36, Lpl, Ldlr, Fatp1, Fatp2, Fatp3, Fatp4, and Fatp5* (Fig. 7). Chronic alcohol drinking significantly enhanced *Srebf1* expression and the mRNA level of *Cd36* and *Lpl, Fatp4* related to lipid uptake. However, licorice supplement significantly prevented the induction of these genes expression.

## Discussion

Although the mechanisms of the induction of fatty liver by alcohol appear to be complicated, accumulating lines of evidence suggest contribution of both oxidative stress and inflammation. On the basis of our recent findings that licorice protects cells against inflammation and oxidative stress [16], we hypothesized that licorice would alleviate alcohol-induced fatty liver injury.

In mice fed a standard Lieber–DeCarli alcohol diet for 4 weeks, hepatic triglyceride levels increased and GSH content decreased with concomitant increases in serum ALT and AST activities, triglycerides, and TNF-α. Supplementation of the alcohol diet with licorice for the same time period significantly reversed the changes in liver injury markers and effectively abrogated fat accumulation. Thus, we suggest that the hepatoprotective effect of licorice is associated with an augmentation of antioxidant defense and anti-inflammatory response.

GSH, a thiol-containing tripeptide, plays a major antioxidant and detoxification role in the liver. Alcohol increases the levels of intracellular reactive oxygen species and depletes mitochondrial GSH, and therefore induces oxidative stress [18]. Although the contribution of oxidative injury to the development of alcoholic fatty liver remains to be elucidated, enhancement of antioxidant capacity using some compounds ameliorates alcoholic fatty liver [19–22]. In line with these results, overexpression of superoxide dismutase prevents the accumulation of lipid droplets in hepatocytes, whereas double knockout of glutathione peroxidase-1 and catalase aggravates alcohol-induced liver injury [23–25]. Our results thus indicate that an improvement in the antioxidant capacity in alcohol-fed mice via recovery of the hepatic GSH pool could make licorice valuable in the treatment of alcoholic liver disease.

Direct inflammatory and cytotoxic effects of TNF-α in alcoholic liver disease are well characterized. Chronic drinking of alcohol increases the level of bacterial endotoxin, which stimulates resident liver macrophages to produce free radicals and cytokines [26]. NADPH oxidase plays critical roles in the generation of oxidants in resident liver macrophages after alcohol intake. Activation of NF-kB by oxidant generation leads to an increase in the TNF-α level, which causes tissue injury [27]. Moreover, TNF-α is suggested to induce lipolysis in adipose tissue followed eventually by fatty liver. Earlier studies showed that TNF-α causes free fatty acid release from adipocytes, stimulates lipogenesis in the liver, and inhibits β-oxidation of free fatty acids [28–30]. Moreover, in a more recent report, TNF-α was suggested to increase intrahepatic fat deposition by affecting hepatic lipogenic metabolism that involves SREBP-1c [31]. Indeed, TNFR1 knockout almost completely blocks the development of alcohol-induced fatty liver [32] . In agreement with these reports, the present study demonstrated that licorice significantly inhibited up-regulation of *Srebf1* by chronic alcohol drinking. Importantly, increase of gene expression involved in lipid uptake such as *Cd36, Lpl,* and *Fatp4* is also effectively reduced by licorice treatment. Considering the importance of TNF-α in the development of alcoholic fatty liver, suppression of TNF-α secretion by licorice may contribute to its overall preventive effect in alcoholic liver injury.

## Conclusion

We found that licorice is effective in preventing alcoholic fatty liver in mice. An important issue in the management of alcoholic liver disease is the progression of simple fat accumulation to alcoholic hepatitis. Licorice treatment restored hepatic GSH content and inhibited TNF-α secretion, and also inhibited lipid accumulation in the liver of chronic alcohol-fed mice. Therefore, licorice is a promising candidate to prevent the progression of alcoholic liver injury, which probably acts by enhancing anti-oxidative and anti-inflammatory capacity.

## Additional file

Additional file 1: Table S1. List of murine primers used for real time RT-PCR. (DOCX 17 kb)
