# Supplementary material for: Hepatoprotective effect of licorice, the root of Glycyrrhiza uralensis Fischer, in alcohol-induced fatty liver disease
Source: BMC Complement Altern Med. 2016 Jan 22;16:19. doi: 10.1186/s12906-016-0997-0 (PMC4722619; doi:10.1186/s12906-016-0997-0)
Supplement: Additional file 1: Table S1. — List of murine primers used for real time RT-PCR. (DOCX 17 kb) [file 12906_2016_997_MOESM1_ESM.docx]

**Additional file 1: Table S1. List of murine primers used for real time RT-PCR**

| **Symbol** | **Full name** | **Primer sequence (5’-3’)** |  |
| --- | --- | --- | --- |
|  |  | **Forward** | **Reverse** |
| *Srebf1* | sterol regulatory element binding transcription factor 1 | GATGTGCGAACTGGACACAG | CATAGGGGGCGTCAAACAG |
| *Fasn* | fatty acid synthase | ACCTCTCCCAGGTGTGTGAC | CCTCCCGTACACTCACTCGT |
| *Mttp* | microsomal triglyceride transfer protein | CTCTTGGCAGTGCTTTTTCTCT | GAGCTTGTATAGCCGCTCATT |
| *Apob* | apolipoprotein B | TTGGCAAACTGCATAGCATCC | TCAAATTGGGACTCTCCTTTAGC |
| *Cd36* | Cd36 antigen | CCTTGGCAACCAACCACAAA | ATCCACCAGTTGCTCCACAC |
| *Lpl* | lipoprotein lipase | ATCGGAGAACTGCTCATGATGA | CGGATCCTCTCGATGACGAA |
| *Ldlr* | low density lipoprotein receptor | TTCAGTGCCAATCGACTCAC | TGTGACCTTGTGGAACAGGA |
| *Fatp1* | fatty acid transporter, member 1 | CGCTTTCTGCGTATCGTCTG | GATGCACGGGATCGTGTCT |
| *Fatp2* | fatty acid transporter, member 2 | GGTATGGGACAGGCCTTGCT | GGGCATTGTGGTATAGATGACATC |
| *Fatp3* | fatty acid transporter, member 3 | AGTGCCAGGGATTCTACCATC | GAACTTGGGTTTCAGCACCAC |
| *Fatp4* | fatty acid transporter, member 4 | GATGGCCTCAGCTATCTGTGA | GGTGCCCGATGTGTAGATGTA |
| *Fatp5* | fatty acid transporter, member 5 | CTACGCTGGCTGCATATAGATG | CCACAAAGGTCTCTGGAGGAT |
| *18S* | 18S ribosomal RNA | CAGCCACCCGAGATTGAGCA | TAGTAGCGACGGGCGGTGTG |
